# Percent body fat was negatively correlated with Testosterone levels in male

**DOI:** 10.1371/journal.pone.0294567

**Published:** 2024-01-03

**Authors:** Hailu Ma, Juan Sun, Xueyan Wu, Jiangfeng Mao, Qin Han

**Affiliations:** 1 Department of Endocrinology, Peking Union Medical College Hospital, Chinese Academy of Medical Sciences and Peking Union Medical College, Beijing, China; 2 Department of General Surgery, Peking Union Medical College Hospital, Chinese Academy of Medical Sciences and Peking Union Medical College, Beijing, China; 3 Beijing Key Laboratory, Institute of Basic Medical Sciences of the Chinese Academy of Medical Sciences, School of Basic Medicine, Peking Union Medical College, Peking Union Medical College Hospital, Center of Excellence in Tissue Engineering of Chinese Academy of Medical Sciences, Beijing, China; Shahjalal University of Science and Technology, BANGLADESH

## Abstract

**Background:**

Lower testosterone levels in men have been consistently associated with metabolic abnormalities, particularly obesity. This study aims to investigate the relationship between testosterone and obesity by analyzing the correlation between testosterone levels and body fat percentage using data from the NHANES (National Health and Nutrition Examination Survey) database.

**Methods:**

The study included a total of 5959 participants from the NHANES 2011–2016. Multivariable linear regression models were used to assess the association between testosterone levels and body composition parameters, including total percent fat (TPF), android percent fat (APF), gynoid percent fat (GPF), android to gynoid ratio (A/G), and lean mass percent (LMP). Subgroup analyses stratified by sex were conducted using multivariable linear regression. To account for potential non-linear relationships, fitted smoothing curves and generalized additive models were utilized. A separate analysis of participants with a BMI ≥ 30 kg/m^2^ was conducted to validate the conclusions.

**Result:**

Among males, testosterone levels showed a significant negative correlation with TPF (β = -11.97, P <0.0001), APF (β = -9.36, P<0.0001), GPF (β = -10.29, P <0.0001), and A/G (β = -320.93, P<0.0001), while a positive correlation was observed between LMP and testosterone levels (β = 12.62, P<0.0001). In females, a contrasting pattern emerged in the relationship between testosterone and body fat, but no significant correlation was found between testosterone and body composition in obese women.

**Conclusions:**

The findings of this study support a negative association between body fat and testosterone levels in males.

## Introduction

Obesity is a metabolic disease characterized by an excess of adipose tissue, with a BMI (Body Mass Index) of ≥30kg/m^2^. The prevalence of obesity has significantly increased worldwide, accompanied by a rise in many health problems such as cardiovascular disease, type 2 diabetes, hypertension, musculoskeletal disorders, respiratory diseases, and certain cancers [1]. These diseases can cause disability and premature death, severely affecting quality of life and life expectancy. In recent years, the relation between obesity and hypogonadism was becoming a hot topic. Extensive research has reported the differing contributions of testosterone to obesity in males and females。

Testosterone is one of the most important representatives of the androgen hormone family. It is mainly synthesized in the testes and to a lesser extent in the ovaries and adrenal glands of females. Testosterone can regulate the development and function of male and female reproductive organs by combining with estrogen, and play an important role in other organs of the body. Testosterone can also affect muscle quality, fat distribution, and metabolic status by regulating protein and fat metabolism [2]. In males, testosterone deficiency can lead to increased fat deposition, fat synthesis, and fat cell proliferation, as well as decreased skeletal muscle mass and basal metabolic rate, thereby exacerbating weight gain, increased body fat percentage, and metabolic syndrome, among other issues [3–5].

However, these studies have been limited by small sample sizes, which may limit their generalizability and statistical power. Therefore, in this study, we aim to investigate the relationship between testosterone levels and obesity using a large dataset from the NHANES (National Health and Nutrition Examination Survey) database. Specifically, we aim to examine the effects of testosterone on body composition, as measured by DXA (Dual-energy X-ray absorptiometry), in both sexes. The results of this study may help to improve our understanding of the role of testosterone in obesity and its associated health risks, and may have important implications for the development of effective interventions for obesity management.

## Materials and methods

### Study population

**Fig 1:**
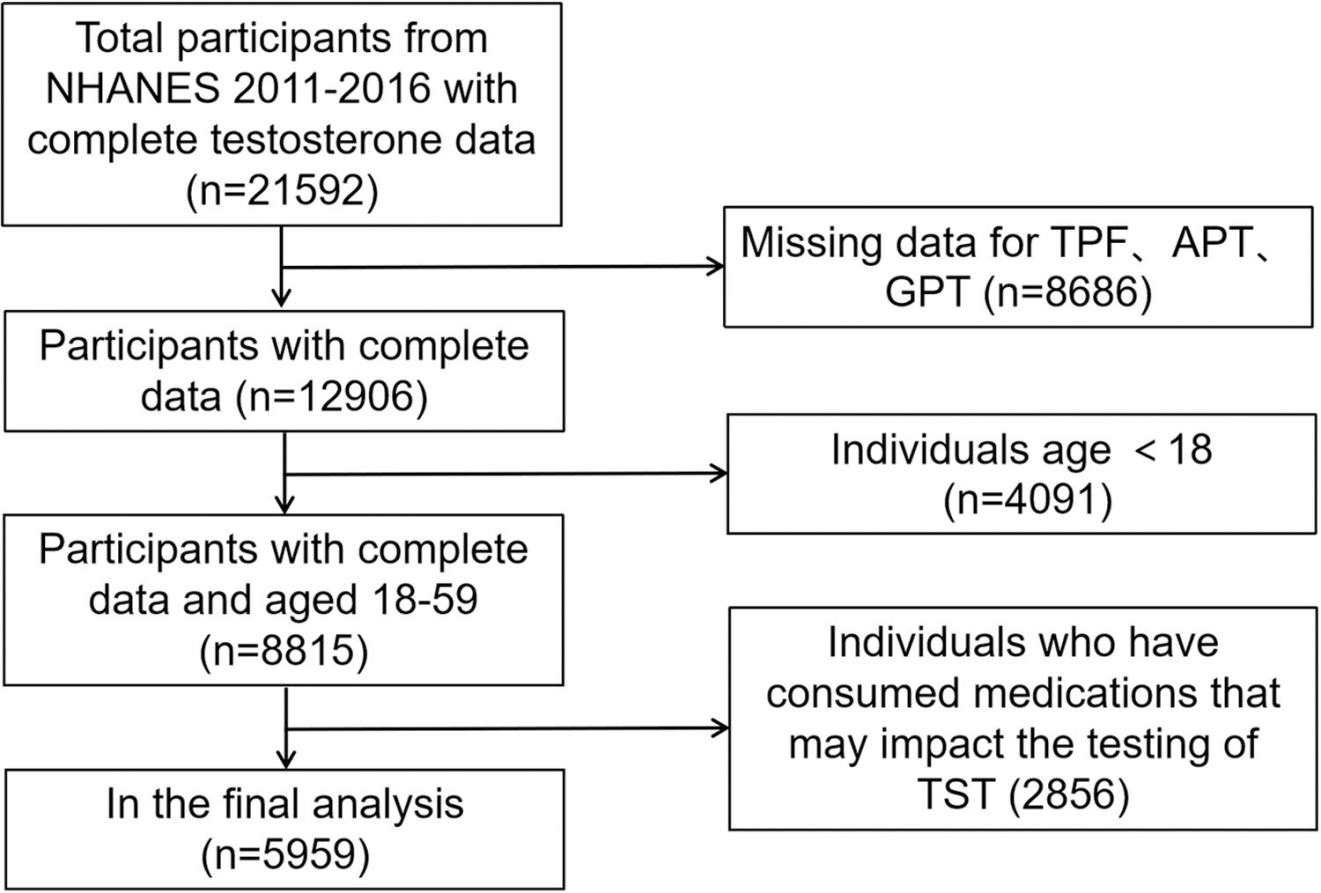
Flow chart of sample selection from the NHANES 2011–2016.

The data for this study were obtained from the National Health and Nutrition Examination Survey (NHANES) conducted by the Centers for Disease Control and Prevention (CDC) and the National Center for Health Statistics (NCHS) between 2011 and 2016. Participant inclusion and exclusion criteria are illustrated in Fig 1. A total of 21,592 participants with complete testosterone data were initially identified. We excluded 8,686 participants with incomplete body composition data, 4,091 participants under the age of 18 and 2,856 participants who had taken medications known to potentially affect testosterone levels (e.g., birth control pills, estrogen, aldactone). Consequently, our analysis focused on 5959 aged 18–59 years old, who had complete data and underwent DXA scans. The National Center for Health Statistics Research Ethics Review Board has thoroughly examined and endorsed the NHANES study and its protocols. Prior to participating, all individuals willingly gave their informed consent by signing the consent form. The specific NHANES cycle for which we obtained approval is a continuation of Protocol #2011–17. For further details, please visit the CDC’s website at: [insert URL: https://www.cdc.gov/nchs/nhanes/irba98.htm].

### Variables

The exposure variables in this study included total percent fat (TPF), android percent fat (APF), gynoid percent fat (GPF), android to gynoid ratio (A/G) and lean mass percent (LMP). These variables were quantified using the Hologic Discovery model A densitometers (Hologic, Inc., Bedford, Massachusetts) with software version Apex 3.2. The measurements were conducted by trained and certified radiology technologists. TPF was determined by calculating the ratio of total fat mass to total fat and lean mass. The android area was defined as the lower trunk region bounded by two lines: the lower pelvic horizontal cut line and a line automatically placed above it. The upper gynoid line was positioned 1.5 times of the height of android region below the pelvic line, while the lower gynoid line was positioned to have a distance between the two gynoid lines twice the height of the android region [6]. The A/G ratio was derived from the ratio of APF to GPF,and LMP was calculated by dividing the total lean mass by the sum of total fat and lean mass. The placement of all these lines was automatically determined by the Hologic software.

The testosterone levels, which served as the outcome variable, were measured using the isotope dilution liquid chromatography tandem mass spectrometry (ID-LC-MS/MS) method. This method was routinely developed by the CDC. The specific details regarding the testosterone measurements can be found in the Laboratory Data section, specifically the Sex Steroid Hormone chapter for the 2013–2014 and 2015–2016 cycles, as well as the Total Testosterone chapter for the 2011–2012 cycle.

Additional factors considered in this study encompassed demographic characteristics such as age, gender, ethnicity, and BMI (Body Mass Index), as well as socioeconomic factors including the ratio of family income to poverty. Physical measurements such as arm circumference and waist circumference were also taken into account. Moreover, the presence of hypertension, hypercholesteremia, and diabetes, as well as smoking status (defined as having smoked at least 100 cigarettes in one’s lifetime), vigorous work activity, and various laboratory markers (including cholesterol levels, triglycerides, HDL cholesterol, LDL cholesterol, creatinine, blood urea nitrogen, and uric acid) were included as covariates. Detailed information pertaining to these variables can be accessed on the official NHANES website.

### Statistical analyses

The demographic characteristics of the individuals participating in the study were reported as mean ± standard deviation (SD) for continuous variables with a normal distribution, and as Median (Q1, Q3) for variables without a normal distribution. Categorical variables were expressed as percentages. Multivariable linear regression models were utilized to determine the association between body composition and testosterone levels. Model 1 did not include any covariates, while Model 2 included adjustments for age, sex, and race. Model 3 built upon Model 2 by additionally adjusting for hypertension, diabetes, hypercholesteremia, smoking status, and vigorous work activity. Additionally, a subgroup analysis stratified by sex was conducted using multivariable regression. To account for any potential nonlinear relationship between body composition and testosterone levels, smoothing techniques and generalized additive models were employed. All statistical analyses were carried out using R (http://www.Rproject.org) and EmpowerStats (http://www.empowerstats.com). A significance level below P < 0.05 was deemed to indicate statistical significance.

## Results

A total of 5959 participants with complete data were included in this study. Table 1 presents the weighted characteristics of the study samples based on sex. There are noticeable disparities between males and females in terms of baseline characteristics. Firstly, males exhibit significantly higher levels of testosterone when compared to females (p<0.001). Secondly, despite males having higher overall energy intake and consuming more protein, carbohydrates, and fat than females, they possess lower measures of TPF, APF, and GPF in comparison to females. Conversely, males exhibit higher A/G ratio, LMP, arm circumference, and waist circumference, all with p-values <0.001. Furthermore, male BMI is lower than that of females (p<0.01). Moreover, females display lower proportions of high blood pressure, hyperlipidemia, smoking, and engagement in physical activity. There is no significant difference between the two genders in terms of diabetes prevalence.

**Table 1:** Weighted characteristics of study samples based on sex.

	Males (n = 4434)	Females (n = 1525)	P-value
Testosterone (ng/dL)	403.32 (304.00, 525.34)	22.30 (15.50, 30.80)	<0.001
Total percent fat (%)	27.09 ± 6.07	38.67 ± 6.33	<0.001
Android percent fat (%)	31.36 ± 8.32	38.68 ± 8.31	<0.001
Gynoid percent fat (%)	28.63 ± 5.64	42.29 ± 5.28	<0.001
Android to gynoid ratio	1.09 ± 0.18	0.91 ± 0.15	<0.001
Lean mass percent (%)	69.87 ± 5.64	58.48 ± 5.85	<0.001
Age (years)	38.26 ± 12.28	35.73 ± 12.26	<0.001
Ratio of family income to poverty	2.11 (1.06–4.12)	1.49 (0.82–3.17)	<0.001
BMI (kg/m^2^)	28.41 ± 5.84	28.99 ± 7.68	<0.01
Arm circumference (cm)	34.39 ± 4.31	31.98 ± 5.64	<0.001
Waist circumference (cm)	99.01 ± 15.41	94.86 ± 17.59	<0.001
Cholesterol (mmol/L)	4.92 ± 1.08	4.76 ± 0.98	<0.001
Triglycerides (mmol/L)	1.42 (0.91–2.33)	1.12 (0.74–1.71)	<0.001
HDL Cholesterol (mmol/L)	1.23 ± 0.34	1.44 ± 0.39	<0.001
LDL Cholesterol (mmol/L)	2.97 ± 0.90	2.81 ± 0.88	<0.001
Creatinine (μmol/L)	83.10 (74.26–92.82)	60.11 (52.16–68.07)	<0.001
Blood urea nitrogen (mmol/L)	4.64 (3.57–5.36)	3.93 (3.21–4.64)	<0.001
Serum uric acid (μmol/L)	358.48 ± 71.73	273.42 ± 62.90	<0.001
Energy intake (kcal)	2510.07 ± 903.12	1812.64 ± 703.12	<0.001
Protein intake (g)	99.50 ± 41.28	70.31 ± 29.61	<0.001
Carbohydrate intake (g)	294.03 ± 118.90	226.34 ± 92.84	<0.001
Fat intake (g)	95.44 ± 42.03	69.20 ± 33.64	<0.001
Race (%)			<0.001
Hispanic	18.25	27.34	
Non-Hispanic White	62.18	44.53	
Non-Hispanic Black	10.55	13.10	
Others	9.02	15.03	
Hypertension (%)			<0.001
Yes	22.81	17.33	
No	77.19	82.67	
Hypercholesteremia(%)			<0.001
Yes	27.47	17.35	
No	72.53	82.65	
Diabetes (%)			0.34
Yes	8.90	8.05	
No	91.10	91.95	
Smoking status (%)			<0.001
Yes	45.76	27.00	
No	54.24	73.00	
Vigorous work activity (%)			<0.001
Yes	33.27	14.78	
No	66.73	85.22	

**Notes:** Mean ± SD and median (Q1, Q3) for continuous variables, p-values were obtained using the weighted linear regression model and Kruskal-Wallis rank sum test. % for categorical variables and P value was calculated by weighted chi-square test.

### Correlation between body composition and testosterone

After adjusting for age, sex, race, hypertension, diabetes, hypercholesteremia, smoking status, and vigorous work activity in model 3, significant negative correlations were observed between TPF, APF, GPF, A/G ratio, and testosterone levels. Conversely, a significantly positive association was found between LMP and testosterone levels. Subgroup analyses stratified by sex revealed a similar trend in males, while in females, a reverse correlation pattern was observed, with testosterone levels positively associated with body fat percentage and negatively associated with muscle mass, as presented in Table 2.

**Table 2:** The association between body composition and testosterone (ng/dL).

	Model 1	Model 2	Model 3
	β (95% CI) P value	β (95% CI) P value	β (95% CI) P value
**Total**			
TPF (%)	-18.84 (-19.41, -18.27) <0.001	-9.25 (-9.90, -8.61) <0.001	-9.39 (-10.01, -8.77) <0.001
APF (%)	-12.84 (-13.40, -12.27) <0.001	-7.33 (-7.81, -6.85) <0.001	-7.44 (-7.90, -6.98) <0.001
GPF (%)	-18.91 (-19.46, -18.36) <0.001	-8.36 (-9.06, -7.66) <0.001	-8.42 (-9.10, -7.73) <0.001
A/G	76.68 (46.61, 106.75) <0.001	-270.4 (-294.44, -246.37) <0.001	-283.28 (-306.52, -260.03) <0.001
LMP (%)	20.11 (19.51, 20.70) <0.001	9.78 (9.09, 10.47) <0.001	9.94 (9.26, 10.61) <0.001
**Sex**			
** Males**			
TPF (%)	-12.44 (-13.21, -11.66) <0.001	-11.97 (-12.80, -11.15) <0.001	-12.09 (-12.89, -11.29) <0.001
APF (%)	-9.52 (-10.07, -8.96) <0.001	-9.36 (-9.97, -8.75) <0.001	-9.46 (-10.05, -8.87) <0.001
GPF (%)	-10.72 (-11.58, -9.85) <0.001	-10.29 (-11.18, -9.40) <0.001	-10.35 (-11.22, -9.48) <0.001
A/G	-337.36 (-364.02, -310.71)<0.01	-320.93 (-351.37, -290.5) <0.01	-337.24 (-366.67, -307.82) <0.001
LMP (%)	13.17 (12.33, 14.00) <0.001	12.62 (11.73, 13.51) <0.001	12.76 (11.90, 13.63) <0.001
** Females**			
TPF (%)	0.08 (-0.05, 0.21) 0.24	0.19 (0.06, 0.33) <0.01	0.20 (0.07, 0.33) <0.01
APF (%)	0.08 (-0.01, 0.18) 0.09	0.15 (0.05, 0.25) 0.0043	0.17 (0.07, 0.26) <0.01
GPF (%)	0.08 (-0.08, 0.24) 0.31	0.15 (-0.01, 0.31) 0.06	0.15 (-0.00, 0.30) 0.06
A/G	4.65 (-0.94, 10.24) 0.10	7.83 (2.00, 13.67) <0.01	8.93 (3.37, 14.49) <0.01
LMP (%)	-0.09 (-0.23, 0.05) 0.22	-0.21 (-0.35, -0.06) <0.01	-0.22 (-0.36, -0.08) <0.01

**Notes:** No covariate was adjusted in Model 1. Model 2 indicates that analysis was adjusted for age, sex, and race. Model 3 indicates model 2 adjustment plus the adjustment for hypertension, diabetes, hypercholesteremia, smoking status, vigorous work activity.

Sex was not adjusted in the sex-stratified subgroup analyses.

In detail, in the fully adjusted model 3, negative correlations were found between TPF, APF, GPF, A/G ratio, and testosterone levels in males. Conversely, a positive relationship was observed between LMP and testosterone level. Specifically, testosterone decreased by 11.97 ng/dL with every 1% increase in TPF, by 9.36 ng/dL with every 1% increase in APF, and by 10.29 ng/dL with every 1% increase in GPF. Furthermore, for every unit increase in A/G ratio, testosterone levels decreased by 320.93 ng/dL, while testosterone levels increased by 12.62 ng/dL for every 1% increase in LMP.

For females, there were a positive correlations between TPF, APF, GPF, A/G ratio with testosterone level. A negative correlation was found between LMP and testosterone levels. Specifically, when TPF increased by 1%, testosterone levels increased by 0.19 ng/dL. For every 1% increase in APF, testosterone increased by 0.15ng/dL, and for every 1% increase in GPF, testosterone levels decreased by 0.15 ng/dL. When A/G increased by 1, testosterone increased by 7.83 ng/dL. Additionally, testosterone decreased by 0.21 ng/dL for every 1% increase in LMP.

Furthermore, we conducted an analysis on the population with a BMI ≥ 30, which is classified as obese, and observed consistent findings with those reported earlier in males. However, in females, no significant correlations were observed between testosterone levels and TPF,APF, GPF, or LMP. These results are presented in Table 3.

**Table 3:** The association between body composition and testosterone (ng/dL) of participants with BMI ≥ 30.

	Model 1	Model 2	Model 3
	β (95% CI) P value	β (95% CI) P value	β (95% CI) P value
**Total**			
TPF (%)	-16.88 (-17.79, -15.97) <0.001	-5.57 (-6.70, -4.44) <0.001	-5.32 (-6.47, -4.16) <0.001
APF (%)	-14.51 (-15.65, -13.37) <0.001	-5.04 (-6.03, -4.04) <0.001	-4.82 (-5.83, -3.80) <0.001
GPF (%)	-14.80 (-15.68, -13.92) <0.001	-2.88 (-3.99, -1.77) <0.001	-2.86 (-3.98, -1.73) <0.001
A/G	355.57 (305.74, 405.40) <0.001	-123.59 (-165.31, -81.87) <0.001	-111.92 (-154.41, -69.43) <0.001
LMP (%)	17.56 (16.62, 18.50) <0.001	5.63 (4.44, 6.82) <0.001	5.37 (4.15, 6.58) <0.001
**Sex**			
** Males**			
TPF (%)	-6.74 (-8.19, -5.29) <0.001	-6.80 (-8.26, -5.33) <0.001	-6.54 (-8.04, -5.04) <0.001
APF (%)	-6.27 (-7.56, -4.98) <0.001	-6.29 (-7.59, -4.99) <0.001	-6.07 (-7.39, -4.74) <0.001
GPF (%)	-3.40 (-4.84, -1.96) <0.001	-3.61 (-5.09, -2.14) <0.001	-3.61 (-5.10, -2.12) <0.001
A/G	-143.20 (-194.59, -91.81) <0.001	-147.85 (-201.21, -94.48) <0.001	-131.90 (-186.31, -77.48) <0.001
LMP (%)	6.81 (5.28, 8.34) <0.001	6.87 (5.32, 8.41) <0.001	6.59 (5.01, 8.17) <0.001
**Females**			
TPF (%)	0.07 (-0.29, 0.43) 0.72	0.14 (-0.22, 0.49) 0.46	0.19 (-0.18, 0.56) 0.31
APF (%)	0.19 (-0.11, 0.49) 0.220	0.18 (-0.12, 0.48) 0.24	0.21 (-0.10, 0.51) 0.18
GPF (%)	0.03 (-0.29, 0.36) 0.85	-0.02 (-0.34, 0.31) 0.92	0.05 (-0.28, 0.38) 0.7736
A/G	10.39 (-4.31, 25.08) 0.17	13.01 (-1.47, 27.48) 0.08	12.00 (-3.02, 27.01) 0.12
LMP (%)	-0.06 (-0.44, 0.32) 0.77	-0.12 (-0.50, 0.26) 0.53	-0.18 (-0.57, 0.21) 0.36

**Notes:** No covariate was adjusted in Model 1. Model 2 indicates that analysis was adjusted for age, sex, and race. Model 3 indicates model 2 adjustment plus the adjustment for hypertension, diabetes, hypercholesteremia, smoking status, vigorous work activity.

Sex was not adjusted in the sex-stratified subgroup analyses.

**Fig 2:**
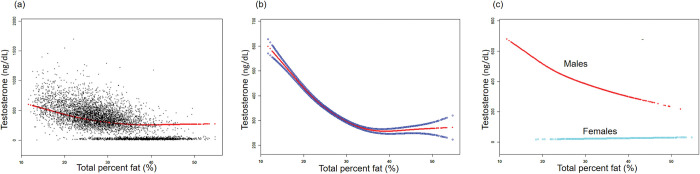
The association between total percent fat (%) and testosterone (ng/dL). **(a)** Each black point represents a sample. **(b)** Solid red line represents the smooth curve fit between variables. Blue bands represent the 95% confidence bands derived from the fit. **(c)** Stratified by sex. Age, sex, race, hypertension, diabetes, hypercholesteremia, smoking status, vigorous work activity were adjusted (c was not sex-adjusted).

**Fig 3:**
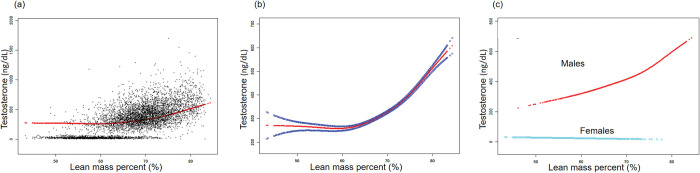
The association between lean mass percent (%) and testosterone (ng/dL). **(a)** Each black point represents a sample. **(b)** Solid red line represents the smooth curve fit between variables. Blue bands represent the 95% confidence bands derived from the fit. **(c)** Stratified by sex. Age, sex, race, hypertension, diabetes, hypercholesteremia, smoking status, vigorous work activity were adjusted (c was not sex-adjusted).

The non-linear relationships between TPF, LMP, and testosterone level were further analyzed using smooth curve fittings and generalized additive models (Figs 2 and 3). Additionally, the results of subgroup analysis stratified by sex are presented in Table 2. The smooth curve fittings between APF, GPF, and testosterone levels were similar to those of TPF with testosterone levels, which are presented in the S1 and S2 Figs.

We also performed subgroup analyses based on the presence of diabetes, which showed a negative correlation between testosterone levels and body fat percentage in both non-diabetic and diabetic population. These results are presented in the S1 Table.

## Discussion

This study revealed notable gender differences in energy intake and body composition. Specifically, it was observed that male participants exhibited higher overall energy intake, while simultaneously presenting lower levels of total protein intake (TPF), amino acid intake (APF), glucose intake (GPF), and body mass index (BMI) in comparison to their female counterparts. Conversely, males displayed higher A/G ratio, lean mass percentage (LMP), arm circumference, and waist circumference as opposed to females. Further investigations revealed, in males, testosterone levels were negatively correlated with TPF, APF, and GPF, and positively correlated with LBM. However in females, there is no significant correlation between testosterone and body composition. These results suggest that testosterone may exert different physiological effects in men and women.

In males, testosterone is known to play a critical role in the regulation of metabolism, and studies have shown that low testosterone levels may lead to obesity. Studies have shown that testosterone deprivation treatment can lead to an increase in visceral, abdominal subcutaneous, and total fat in patients with prostate cancer [7]. Conversely, testosterone supplementation has been shown to decrease abdominal and thigh subcutaneous fat in men with low testosterone levels and high waist circumference [8]. Additionally, weight loss and bariatric surgery in young men have been associated with an increase in total and free testosterone concentrations [9]. These findings suggest bidirectional regulatory effects between serum testosterone and obesity in males.

Obesity may induce male hypogonadism. Adipocytes in obese individuals exhibit elevated levels of aromatase, which catalyzes the conversion of testosterone to estradiol [10]. Estradiol, along with other adipocytokines such as TNF, IL-6, and leptin, can negatively impact testosterone synthesis through a variety of mechanisms, including inhibition of the hypothalamic-pituitary-testicular axis [11], suppression of kisspeptin neuron function [12], and direct inhibition of gonadotropin release by leptin [13]. Conversely, testosterone deficiency can promote the differentiation of pluripotent stem cells into adipocytes, potentially exacerbating obesity [14].

On the other hand, in males, testosterone supplementation can promote lipolysis by increasing expression of norepinephrine and β-adrenergic receptors in adipocytes [15], and inhibit lipid storage by reducing LPL activity on adipocyte surfaces [16]. Additionally, testosterone can reduce expression of PPARγ in preadipocytes, inhibiting their differentiation into adipocytes [14]. Studies have also shown that testosterone supplementation can increase lipid catabolism and mitochondrial biomarkers in adipose tissue, indicating a potential acceleration of energy consumption in adipocytes [17].

Additionally sufficient testosterone concentration is essential for maintaining muscle mass in men, and testosterone deficiency may result in muscle mass loss. In patients with prostate cancer receiving GnRHa therapy for 48 weeks, the muscle mass percentage decreased by 2.7%. Arik Davidyan et al. explored the effect of testosterone on maintaining muscle mass and found that muscle mass in castrated mice significantly decreased [18].

Testosterone supplementation increases muscle mass and strength. Several studies have reported that testosterone therapy increases lean body mass in men, with gains ranging from 1.9 kg to 3.6 kg [19–21], depending on the duration and dose of treatment. Additionally, testosterone therapy has been shown to improve muscle function and gait performance [21]. The mechanism of action for testosterone-induced muscle growth involves hypertrophy of type I and type II fibers and an increase in the number of muscle bundles and satellite cells [22].

The relationship between testosterone levels and body composition in women remains ambiguous. Some studies suggest a positive correlation between testosterone levels and body fat [23, 24], while others indicate no clear correlation between the two [25, 26]. This suggests that the relationship between testosterone levels and body composition in women is complex and may be influenced by factors such as BMI, age, and hormone balance.

Women with pathologically elevated androgen levels, such as those with polycystic ovary syndrome (PCOS), have a high incidence of overweight and obesity, which is associated with insulin resistance and metabolic disorders [27]. The increased activity of steroid-converting enzymes in PCOS patients indicates that adipose tissue function is influenced by these sex steroid hormones at the tissue level. However, the relationship between obesity and high androgen status in the PCOS population is not yet fully established. In most studies of the PCOS population, total testosterone levels were positively correlated with the presence and amount of abdominal obesity [28, 29], but some studies also reported no association between fat accumulation and total testosterone levels [30].

The complex relationship between testosterone levels and female fat distribution remains unclear, mainly because existing mechanism studies have not shown a clear sex difference in testosterone’s regulation of fat metabolism, such as inhibiting adipocyte differentiation, promoting adipocyte lipolysis, and promoting lipid metabolism [31]. In addition, some studies have shown that appropriate testosterone supplementation plays an important role in maintaining female metabolism. Davis et al. found that testosterone replacement therapy improved body composition, including reducing body fat percentage and increasing muscle mass, in women with low testosterone levels [32]. Liang et al. found that higher TT levels were associated with a gradually lower risk of metabolic syndrome in adult women [33].

Therefore, the results of this study contribute to a deeper understanding of the relationship between testosterone and fat distribution, and further exploration of the impact of testosterone levels and female body composition in high androgen-related diseases such as PCOS and hyperandrogenism.

## Strength and limitation

This study has several strengths, including the use of a large and nationally representative sample of US adults with complete data on TPF, LMP, and testosterone levels, and the use of DXA to measure body composition accurately. The large sample size also allowed for sex-specific subgroup analysis, revealing a previously unreported pattern between testosterone and TPF.

However, this study is limited by its cross-sectional design, which cannot establish causality. Additionally, there may be other unmeasured confounding factors that could affect the observed associations, such as natural differences in TPF and LMP between men and women.

## Conclusion

In conclusion, our results showed that lower testosterone levels in males are linked to more total fat percent and less lean mass percent. Instead, there is no significant correlation between testosterone and body composition in females. The underlying mechanisms of the different patterns of this relationship in males and females still need further investigation.

## Supporting information

S1 Fig The association between android percent fat (%) and testosterone (ng/dL). **(a)** Each black point represents a sample. **(b)** Solid red line represents the smooth curve fit between variables. Blue bands represent the 95% confidence bands derived from the fit. **(c)** Stratified by sex. Age, sex, race, hypertension, diabetes, hyperlipidemia, smoking status, vigorous work activity were adjusted (c was not sex-adjusted).
(TIF)

S2 Fig The association between gynoid percent fat (%) and testosterone (ng/dL). **(a)** Each black point represents a sample. **(b)** Solid red line represents the smooth curve fit between variables. Blue bands represent the 95% confidence bands derived from the fit. **(c)** Stratified by sex. Age, sex, race, hypertension, diabetes, hyperlipidemia, smoking status, vigorous work activity were adjusted (c was not sex-adjusted).
(TIF)

S1 Table The association between body composition and testosterone (ng/dL) stratified by diabetes status. **Notes:** No covariate was adjusted in Model 1. Model 2 indicates that analysis was adjusted for age, sex, and race. Model 3 indicates model 2 adjustment plus the adjustment for hypertension, hyperlipidemia, smoking status, vigorous work activity.
(DOC)
