# Supplementary figures and images for: Percent body fat was negatively correlated with Testosterone levels in male
Source: PLoS One. 2024 Jan 3;19(1):e0294567. doi: 10.1371/journal.pone.0294567 (PMC10763932; doi:10.1371/journal.pone.0294567)

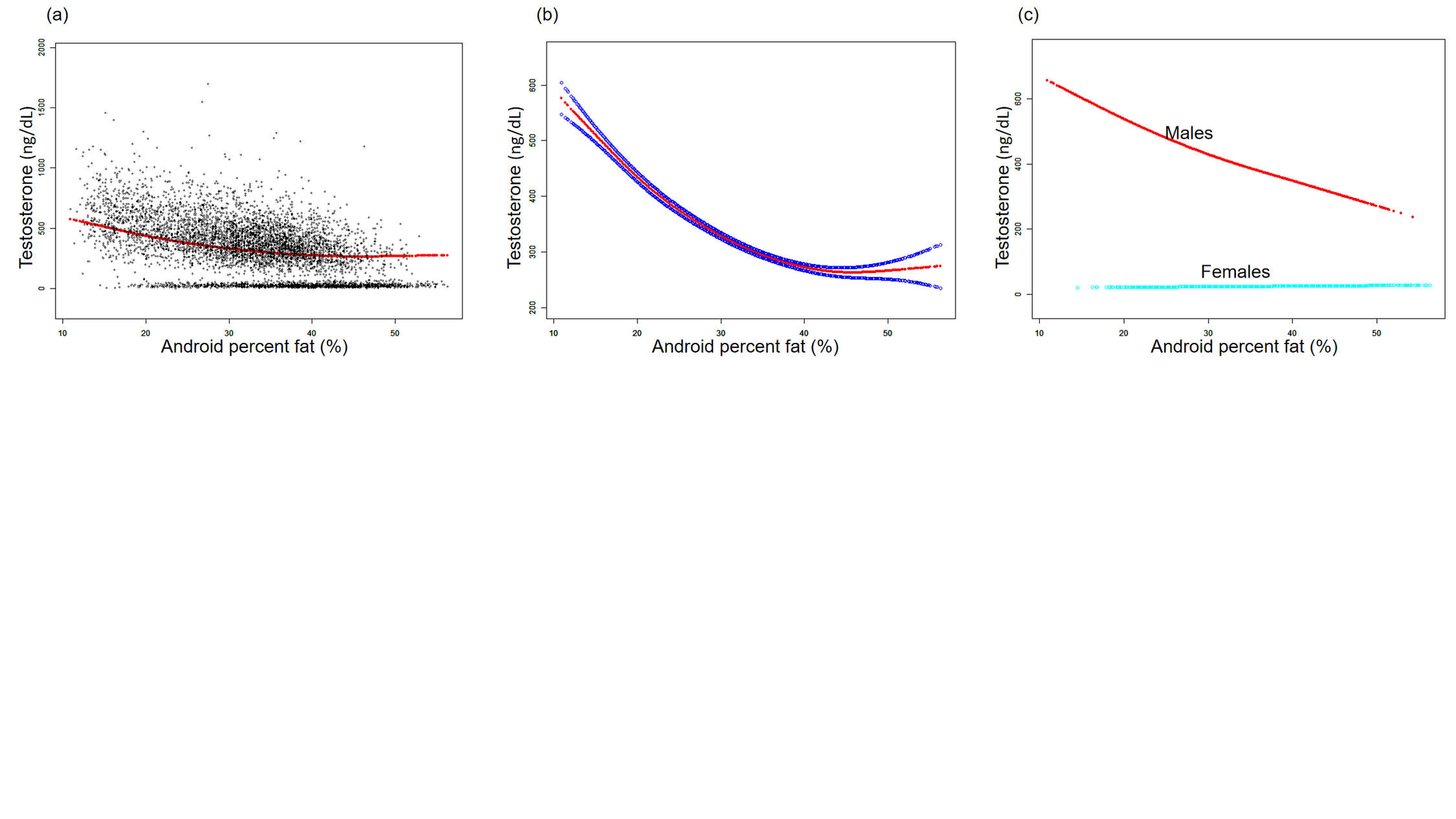

Supplement: S1 Fig — (a) Each black point represents a sample. (b) Solid red line represents the smooth curve fit between variables. Blue bands represent the 95% confidence bands derived from the fit. (c) Stratified by sex. Age, sex, race, hypertension, diabetes, hyperlipidemia, smoking status, vigorous work activity were adjusted (c was not sex-adjusted). (TIF) [file pone.0294567.s001.tif]

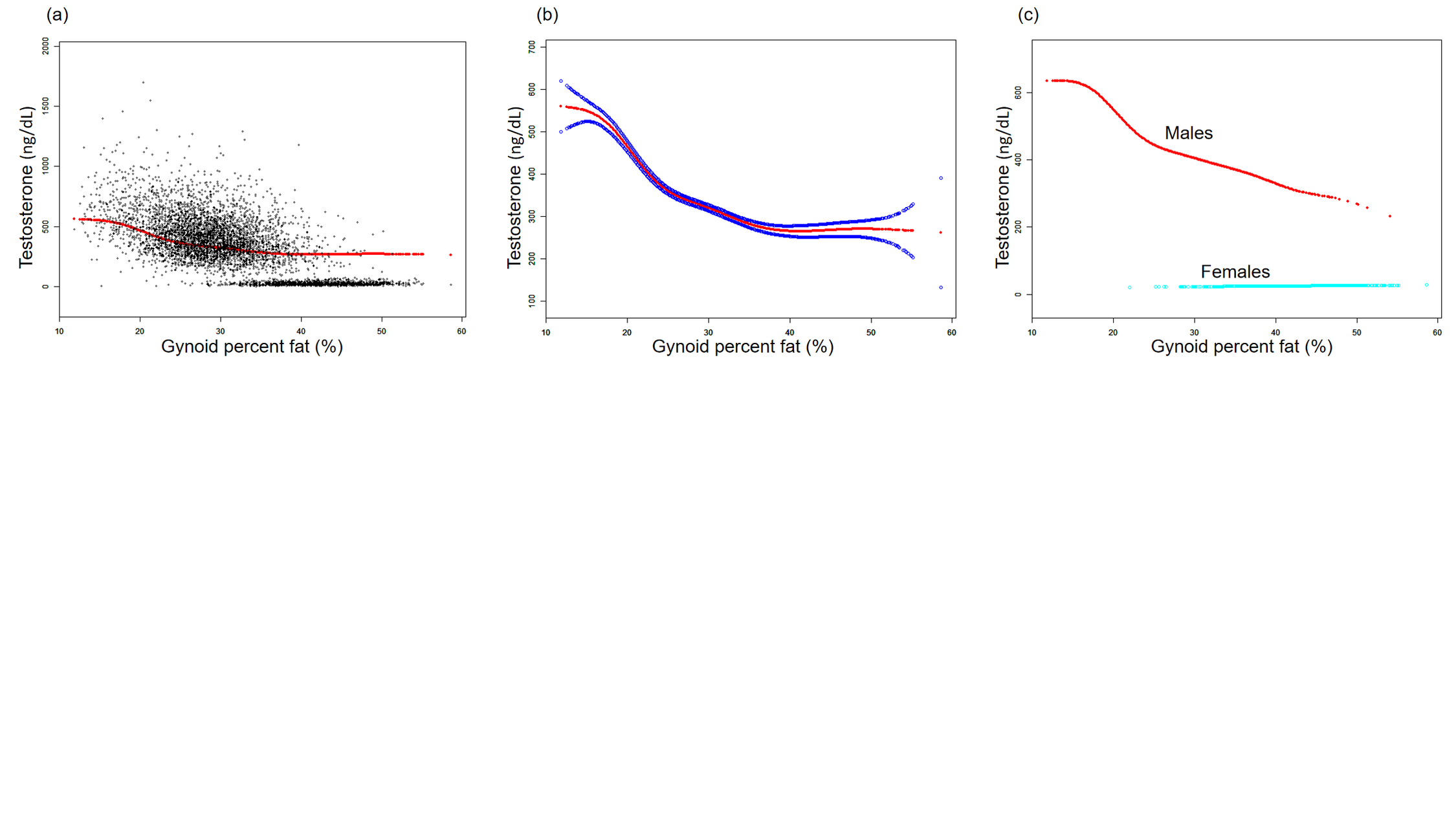

Supplement: S2 Fig — (a) Each black point represents a sample. (b) Solid red line represents the smooth curve fit between variables. Blue bands represent the 95% confidence bands derived from the fit. (c) Stratified by sex. Age, sex, race, hypertension, diabetes, hyperlipidemia, smoking status, vigorous work activity were adjusted (c was not sex-adjusted). (TIF) [file pone.0294567.s002.tif]
