# Supplementary material for: Percent body fat was negatively correlated with Testosterone levels in male
Source: PLoS One. 2024 Jan 3;19(1):e0294567. doi: 10.1371/journal.pone.0294567 (PMC10763932; doi:10.1371/journal.pone.0294567)
Supplement: S1 Table — Notes: No covariate was adjusted in Model 1. Model 2 indicates that analysis was adjusted for age, sex, and race. Model 3 indicates model 2 adjustment plus the adjustment for hypertension, hyperlipidemia, smoking status, vigorous work activity. (DOC) [file pone.0294567.s003.doc]

**S1 Table.** The association between body composition and testosterone (ng/dL) stratified by diabetes status.

|  | Model 1  β (95% CI) P value | Model 2  β (95% CI) P value | Model 3  β (95% CI) P value |
| --- | --- | --- | --- |
| **Diabetes** | | | |
| TPF (%) | -18.17 (-19.82, -16.52) <0.001 | -11.33 (-13.35, -9.31) <0.0001 | -11.02 (-13.07, -8.97) <0.001 |
| APF (%) | -15.62 (-17.39, -13.85) <0.001 | -9.76 (-11.39, -8.13) <0.0001 | -9.45 (-11.09, -7.81) <0.001 |
| GPF (%) | -17.38 (-19.13, -15.62) <0.001 | -8.91 (-11.15, -6.68) <0.0001 | -8.43 (-10.71, -6.15) <0.001 |
| A/G | 72.04 (-21.64, 165.71) 0.13 | -246.37 (-319.11, -173.64) <0.001 | -256.09 (-328.63, -183.55) <0.001 |
| LMP (%) | 19.24 (17.51, 20.96) <0.001 | 12.00 (9.83, 14.17) <0.0001 | 11.64 (9.43, 13.84) <0.001 |
| **Non-diabetes** | | | |
| TPF (%) | -18.95 (-19.55, -18.34) <0.001 | -9.16 (-9.82, -8.50) <0.001 | -9.12 (-9.79, -8.44) <0.001 |
| APF (%) | -12.68 (-13.28, -12.09) <0.001 | -7.23 (-7.72, -6.75) <0.01 | -7.19 (-7.69, -6.69) <0.001 |
| GPF (%) | -18.98 (-19.56, -18.40) <0.001 | -8.26 (-8.98, -7.54) <0.0001 | -8.28 (-9.02, -7.54) <0.001 |
| A/G | 94.03 (61.87, 126.19) <0.001 | -281.52 (-306.15, -256.88) <0.001 | -272.12 (-297.58, -246.67) <0.001 |
| LMP (%) | 20.22 (19.58, 20.85) <0.001 | 9.68 (8.97, 10.39) <0.001 | 9.63 (8.90, 10.35) <0.001 |

**Notes:** No covariate was adjusted in Model 1. Model 2 indicates that analysis was adjusted for age, sex, and race. Model 3 indicates model 2 adjustment plus the adjustment for hypertension, hyperlipidemia, smoking status, vigorous work activit
